# Study of recent and future trends in place of death in Belgium using death certificate data: a shift from hospitals to care homes

**DOI:** 10.1186/1471-2458-11-228

**Published:** 2011-04-13

**Authors:** Dirk Houttekier, Joachim Cohen, Johan Surkyn, Luc Deliens

**Affiliations:** 1 Vrije Universiteit Brussel, End-of-Life Care Research Group Ghent University & Vrije Universiteit Brussel, Laarbeeklaan 103, 1090 Brussels, Belgium; 2 Vrije Universiteit Brussel, Interface demography, Pleinlaan 2, 1050 Brussels, Belgium; 3 Department of Public and Occupational Health, EMGO Institute for Health and Care Research and Palliative Care Center of Expertise, VU University Medical Center, Van der Boechorststraat 7, 1081 BT Amsterdam, the Netherlands

## Abstract

**Background:**

Since most patients prefer out-of-hospital death, place of death can be considered an indicator of end-of-life care quality. The study of trends in place of death is necessary to examine causes of shifts, to evaluate efforts to alter place of death and develop future policies. This study aims to examine past trends and future projections of place of death.

**Methods:**

Analysis of death certificates (decedents aged ≥ 1 year) in Belgium (Flanders and Brussels Capital region) 1998-2007. Trends in place of death were adjusted for cause of death, sociodemographic characteristics, environmental factors, numbers of hospital beds, and residential and skilled nursing beds in care homes. Future trends were based on age- and sex-specific mortality prognoses.

**Results:**

Hospital deaths decreased from 55.1% to 51.7% and care home deaths rose from 18.3% to 22.6%. The percentage of home deaths remained stable. The odds of dying in a care home versus hospital increased steadily and was 1.65 (95%CI:1.53-1.78) in 2007 compared to 1998. This increase could be attributed to the replacement of residential beds by skilled nursing beds. Continuation of these trends would result in the more than doubling of deaths in care homes and a decrease in deaths at home and in hospital by 2040.

**Conclusions:**

Additional end-of-life care resources in care homes largely explain the decrease in hospital deaths. Care homes will become the main locus of end-of-life care in the future. Governments should provide sufficient skilled nursing resources in care homes to fulfil the end-of-life care preferences and needs of patients.

## Background

Place of death is considered an important indicator of quality of end-of-life care. Death in the usual place of residence is associated with the presence of family and friends, comfort and a feeling of control [1]. Most patients prefer to die at home, but in cases of inadequate pain control or when the burden becomes too great for family caregivers, end-of-life care and death in an institution may be preferred [1,2]. Place of death also has important consequences for health care costs, as end-of-life care in hospitals is more expensive than in care homes or at home [3,4]. Nevertheless, in Belgium and most other countries a majority of patients still die in hospital [5-7]. For reasons of cost and quality of care supporting out-of-hospital death has become an important policy objective[8,9]. In Belgium and elsewhere, different models of palliative care services and options for out-of-hospital death have been developed [9,10], but their effectiveness remains unclear.

Studying trends in place of death is necessary to monitor and gain insight into the reasons for changes to evaluate efforts to alter place of death, and to plan future end-of-life care services and facilities. Many previous studies were restricted to cancer patients [11-15] and only considered trends in home or hospital death although, with ageing of the population in developed countries, other chronic life-limiting conditions will become more important (e.g. dementia related diseases) [16] and end-of-life care in care homes will become a major concern because of the expected increase in care home residents. In most studies, shifts in place of death were only related to shifts in age, sex and underlying cause of death [14,17-23], and shifts in other factors were not taken into account, e.g. in living arrangements, educational attainment, urbanization levels, and available care options. Although instituted in 1985, palliative care services in Belgium have been only gradually integrated into formal health care and reimbursed by healthcare insurance since 1997 [9,24]. Studying trends in place of death from 1998 to 2007 allows discussion of the impact of the growing availability of palliative care services and out-of-hospital end-of-life care options on an important patient outcome.

The aim of this study is to examine past trends between 1998 and 2007 and future trends until 2040 of place of death. The research questions are: 1) How did place of death change between 1998 and 2007 taking into account shifts in underlying cause of death, age, sex, living arrangement, educational attainment, urbanization level and availability of hospital and care home beds? 2) Did place of death change differently for specific subpopulations regarding living arrangement? 3) How would place of death change between now and 2040, based on trends between 1998 and 2007?

## Methods

All deaths in Belgium (Flanders and Brussels Capital Region) aged one year or older in the period 1998-2007 were included (N = 661 773). A subpopulation of patients who died non-suddenly of one or more chronic life-limiting conditions (including cancer, cerebrovascular diseases, dementia, chronic obstructive pulmonary disorders, heart failure, diabetes, Parkinson's disease, chronic kidney disease, chronic liver disease, spinal muscular atrophy and related disorders, multiple sclerosis, neuromuscular disorders, and acquired immunodeficiency syndrome) and were thus eligible for palliative care (palliative subset) was considered in multivariate analysis (n = 354 794) [16].

In Belgium, death certificates contain information about the place and cause of death and the sociodemographic characteristics of the deceased. Information about the place and cause of death is completed by a physician, who certifies the causal chain of diseases starting with the underlying cause of death, which is used in this study. Information about the sociodemographic characteristics (e.g. educational attainment, nationality, marital status, and living arrangements) is provided by the municipal institutions assisted by a relative of the deceased. Information from the death certificates is processed; the causes of death are coded in ICD-10 codes, and both medical and sociodemographic information is checked by a government agency [25]. Approval of an ethics committee to use death certificate data was not required, but in accordance with the Belgian Privacy Act, the use of the data was notified to the Belgian Commission for the Protection of Privacy.

Dependent variable is place of death recoded into four categories: home, hospital, care home and elsewhere. In Belgium, care homes are long-term care facilities, including both homes for the aged and skilled nursing facilities, although most have a mix of beds for residents requiring skilled nursing care on a daily basis (skilled nursing beds) and beds for those not requiring such care (residential beds).

The independent variables are the year of death (1998-2007), the illness of the deceased (underlying cause of death, recoded in six categories: cancer, cardiovascular diseases, respiratory diseases, diseases of the nervous system, stroke, and other using ICD-10 codes), the demographic (sex, and age: 1-64 years, 65-74 years, 75-84 years, ≥85 years) and social situation (educational attainment: no formal or elementary education, lower secondary, higher secondary, higher, and other or unknown), living arrangement (living alone, in a multi-person household or in an institution which is a care home in almost all cases), urbanization level (of the municipality of residence: very strong, strong, average, low or rural) and available hospital, and residential and skilled nursing care home beds (data on the availability of care home beds were provided by the Belgian National Institute for Health and Disability Insurance, data on hospital beds by the Federal Public Service of Health, Food Chain Safety and Environment). The availability of skilled nursing beds and residential beds in care homes was considered per 1000 inhabitants of 65 years or older in the community of residence or in areas of up to six smaller communities (115 areas in Flanders with an average population [year 2000] of 51 654 inhabitants. Brussels Capital Region was considered one area) [26]. The availability of hospital beds was considered per 1000 inhabitants in areas of eight to 45 communities (14 areas in Flanders and Brussels Capital Region with an average population of 492 826)

For use in multivariate analysis, the underlying cause of death (cancer versus non-cancer chronic life-limiting conditions), education (no formal or elementary or lower secondary or unknown/other versus higher secondary or higher), and urbanization (very strong or strong versus moderate or low) were recoded into two categories.

Differences in trends in the total number of deaths (aged ≥1 years) in the period 1998-2007 and the number of deaths in the palliative subset, the categories of cause of death, age, sex, living arrangement, urbanization level, and place of death were tested using χ^2^-test for trend.

Differences in trends in the total number of deaths (aged ≥65 years) at home, in hospital, and in care homes and the number at home, in hospital, and in care homes of the categories of living arrangement were tested using χ^2^-test for trend. Differences in the yearly average availability of hospital, residential and skilled nursing beds were tested using the F-test.

Multivariate analyses were performed separately for those aged 65 years or older residing at home and those residing in care homes who died from chronic life-limiting conditions [16], because of differences in age and conditions in both subpopulations. Unadjusted odds ratios of the association of year of death and place of death were compared with odds ratios adjusted for underlying cause of death, age, sex, educational attainment, living arrangement, urbanization level, and available hospital beds, and residential and skilled nursing beds in care homes. The multivariate analyses were binomial logistic regression analyses with forward stepwise likelihood ratio selection procedure of variables and tested using the Wald-test for individual model parameters and model-χ^2 ^for the whole model. The significance level was set at p = 0.01, given the large amount of data.

To examine which factors possibly adjusted the yearly trends in place of death, both logistic regression models were built up step by step. Starting from the unadjusted odds ratios of each year of death relative to 1998 for home death relative to hospital death and care home death relative to hospital death, we added the underlying cause of death, age, sex, educational attainment and living arrangement of the deceased, and the available residential and nursing beds in care homes and hospital beds step by step, to examine which of these factors adjusted the odds of dying in hospital in each year.

We used age- and sex-specific mortality projections of the Belgian National Planning Bureau for Flanders and Brussels Capital Region based on the population statistics of January 1 2007 to project future trends in place of death. Life expectancy at birth is expected to increase with 8 years both for men and women between 2007 and 2060, and to reach 85.3 years for men and 90.9 years for women [27]. These mortality projections were used to perform linear projections of future proportions and numbers of deaths from 2008 to 2040 in all care settings based on age- and sex-specific proportions of deaths in the different care settings we found in the period 1998-2007. Two scenarios were considered; in the first we examined future trends in place of death starting from the age- and sex- specific distribution in 2007, in the second we considered the age- and sex-specific percentage point changes in all categories of place of death from 1998 to 2007 [18].

All analyses were performed using PASW statistics 17.

## Results

In 1998-2007, 661 773 people (aged ≥1 year) died in Belgium (Flanders and Brussels Capital Region), ranging from 64 560 in 2006 to 68 278 in 2003 (Table 1). The proportion of cancer deaths and deaths from diseases of the nervous system grew over that period, while deaths from cardiovascular diseases and stroke diminished. The proportion of deaths of the palliative subset increased from 54.2% to 55.1%. The proportion of deaths of people aged 75 and over, with higher secondary or unknown education, living alone and in strongly or averagely urbanized places increased, while the opposite was true for deaths of people aged between 65 and 74, having no formal or elementary education, living in a multi-person household, and in very strong urbanized places. During the study period many residential beds in care homes were replaced by skilled nursing home beds. The average number of residential beds per 1000 inhabitants of 65 years or older fell from 56 in 1998 to 36 in 2007 while the average number of skilled nursing home beds rose from 12 to 29. The average number of hospital beds per 1000 inhabitants remained stable.

**Table 1:** Population Characteristics of Deaths (aged ≥1 y) 1998-2007 (N = 661 773)

	1998	1999	2000	2001	2002	2003	2004	2005	2006	2007	P-value
	n, %*, P-value for χ^2 ^for trends and F-test (beds)										
ALL DEATHS	66 481	66 946	66 728	65 555	67 123	68 278	65 050	65 617	64 560	65 435	
CAUSE OF DEATH											
Cancer	27.6%	27.0%	26.9%	27.2%	27.1%	26.4%	27.1%	27.4%	28.1%	28.4%	P < 0.001
Cardiovascular Disease	28.6%	28.1%	28.7%	28.5%	28.1%	27.7%	27.9%	26.7%	25.7%	25.7%	P < 0.001
Respiratory Disease	11.4%	11.5%	11.9%	11.5%	12.1%	12.7%	11.7%	12.4%	11.6%	11.7%	P = 0.004
Disease of Nervous System	2.3%	2.2%	2.3%	2.3%	2.5%	2.9%	2.6%	2.9%	3.3%	3.4%	P < 0.001
Stroke (CVA)	8.9%	9.1%	8.7%	8.8%	8.7%	8.4%	8.6%	8.1%	8.0%	7.9%	P < 0.001
Other	21.1%	22.1%	21.5%	21.8%	21.5%	22.0%	22.1%	22.5%	23.2%	23.0%	P < 0.001
PALLIATIVE SUBSET[16](N = 354 794)	54.2%	53.4%	52.8%	53.2%	53.4%	52.9%	53.3%	53.4%	54.5%	55.1%	P < 0.001
AGE											
1-64 year	17.9%	18.0%	17.8%	17.7%	17.1%	16.5%	16.7%	16.7%	16.8%	17.0%	P < 0.001
65-74 year	21.0%	20.0%	19.9%	19.4%	18.6%	18.3%	17.8%	17.7%	16.8%	16.2%	P < 0.001
75-84 year	30.4%	30.1%	30.1%	31.0%	32.7%	34.3%	35.9%	35.1%	35.2%	34.4%	P < 0.001
85+ year	30.6%	31.9%	32.2%	31.9%	31.5%	30.9%	29.6%	30.5%	31.2%	32.4%	P = 0.2
SEX											
Male	49.9%	49.9%	50.0%	49.9%	49.5%	49.1%	49.6%	49.5%	49.6%	49.7%	P = 0.04
Female	50.1%	50.1%	50.0%	50.1%	50.5%	50.9%	50.4%	50.5%	50.4%	50.3%	P = 0.04
EDUCATIONAL ATTAINMENT											
No formal or elementary	44.3%	44.8%	43.3%	40.7%	39.0%	36.6%	34.7%	33.2%	31.5%	30.0%	P < 0.001
Lower secondary	17.4%	17.4%	17.7%	19.0%	18.9%	17.6%	18.3%	18.8%	18.7%	17.3%	P < 0.001
Higher secondary	10.4%	9.4%	10.2%	9.7%	9.2%	9.0%	9.8%	11.5%	12.8%	13.1%	P < 0.001
Higher	4.5%	4.2%	3.9%	4.1%	4.1%	3.7%	4.1%	4.2%	4.4%	4.5%	P = 0.03
Other or unknown	23.4%	24.2%	24.8%	26.6%	28.8%	33.0%	33.1%	32.3%	32.5%	35.2%	P < 0.001
LIVING ARRANGEMENT											
Living alone	21.6%	21.0%	20.9%	21.4%	21.2%	20.7%	21.6%	22.7%	22.3%	23.4%	P < 0.001
Multi-person household	54.4%	54.7%	54.8%	54.0%	53.1%	53.0%	53.1%	51.6%	52.3%	51.9%	P < 0.001
Care home	24.0%	24.2%	24.3%	24.7%	25.6%	26.2%	25.4%	25.7%	25.4%	24.6%	P < 0.001
URBANIZATION LEVEL											
Very strong	39.3%	39.1%	38.8%	38.4%	38.5%	38.1%	38.1%	37.7%	37.4%	36.4%	P < 0.001
Strong	27.9%	27.9%	28.1%	28.1%	28.3%	28.2%	28.2%	28.5%	28.4%	29.7%	P < 0.001
Average	28.5%	28.7%	28.7%	29.1%	28.8%	29.2%	29.4%	29.4%	29.9%	29.4%	P < 0.001
Low or rural	4.3%	4.3%	4.4%	4.4%	4.4%	4.4%	4.3%	4.5%	4.4%	4.5%	P = 0.12
HEALTH CARE RESOURCES											
Hospital beds/1000	5.5	5.4	5.5	5.6	5.6	5.6	5.5	5.4	5.4	5.3	P = 1
Residential beds in care homes/1000≥ 65 y	56.0	52.9	49.9	46.6	43.3	40.2	38.2	37.7	36.6	35.7	P < 0.001
Skilled nursing beds in care homes/1000≥ 65 y	11.6	14.5	17.5	20.2	22.8	25.4	26.9	26.8	27.8	29.0	P < 0.001

* Presented percentages are column percentages

The proportion of deaths in hospitals decreased from 55.1% in 1998 to 51.7% in 2007 (Table 2). In contrast, deaths in care homes increased from 18.3% to 22.6%. Home deaths fell by 0.5% and the trend of home deaths differed insignificantly from the trend of the total number of deaths.

**Table 2:** Trends in Place of Death (aged ≥ 1 y) 1998-2007 (N = 661 771)

	1998	1999	2000	2001	2002	2003	2004	2005	2006	2007	P-value
	
PLACE OF DEATH	n, %*, P-value for χ^2 ^for trends										
Home	15 311	15 389	15 226	14 919	15 075	15 287	14 912	14 878	14 760	14 726	P = 0.4
	23.0%	23.0%	22.8%	22.8%	22.5%	22.4%	22.9%	22.7%	22.9%	22.5%	
Hospital	36 631	36 283	35 924	34 964	35 227	35 574	34 059	34 085	33 264	33 856	P < 0.001
	55.1%	54.2%	53.8%	53.3%	52.5%	52.1%	52.4%	51.9%	51.5%	51.7%	
Care home	12 161	12 980	13 328	13 375	14 578	15 363	14 209	14 605	14 469	14 792	P < 0.001
	18.3%	19.4%	20.0%	20.4%	21.7%	22.5%	21.8%	22.3%	22.4%	22.6%	
Other	2 378	2 293	2 250	2 297	2 242	2 054	1 870	2 049	2 067	2 061	P < 0.001
	3.6%	3.4%	3.4%	3.5%	3.3%	3.0%	2.9%	3.1%	3.2%	3.1%	

* Presented percentages are column percentages

**Figure 1:**
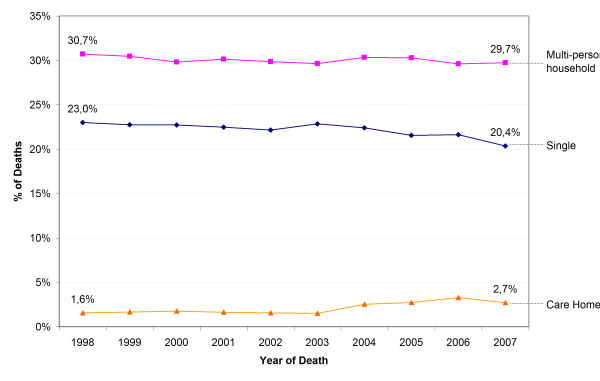
**Trends in home death (aged ≥65 y) by living arrangement**. P-value for χ^2 ^for difference in trend of home death of all categories of living arrangement and the global trend of home death: p < 0.001, except for singles: p = 0.925.

**Figure 2:**
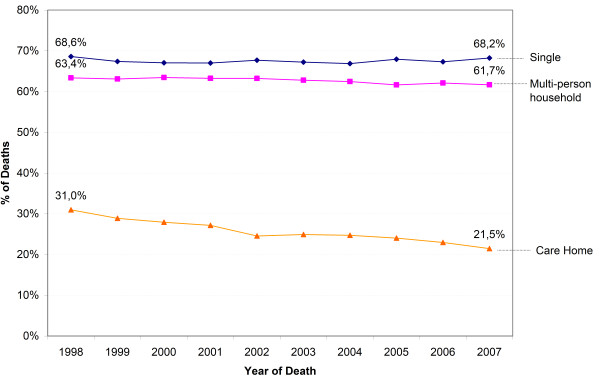
**Trends in hospital death (aged ≥65 y) by living arrangement**. P-value for χ^2 ^for difference in trend of hospital death of all categories of living arrangement and the global trend of hospital death: p < 0.001.

**Figure 3:**
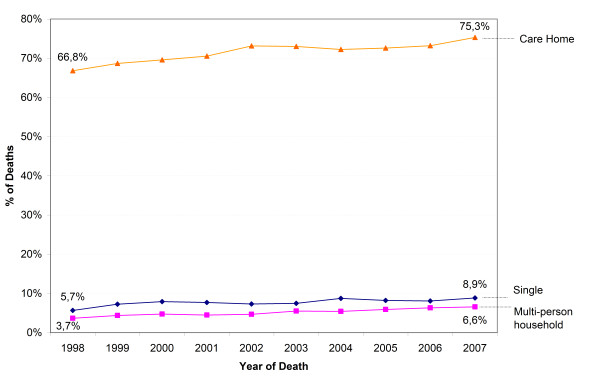
**Trends in care home death (aged ≥65 y) by living arrangement**. P-value for χ^2 ^for difference in trend of care home death of all categories of living arrangement and the global trend of care home death: <0.001

Hospital death rate declined (aged ≥65 years) and care home death rate increased for all categories of living arrangement. The home death rate of people living alone or in multi-person households decreased and that of persons living in care homes increased (Figure 1). The decline of the hospital death rate was largest for those in care homes (from 31.0% to 21.5%) (Figure 2), and was balanced by the increase in the care home death rate (from 66.8% to 75.3%) (Figure 3) and home death rate (from 1.6% to 2.7%). The care home death rate of people living at home increased, both of those living alone (from 5.7% to 8.9%) and in a multi-person household (from 3.7% to 6.6%).

The chances of dying in hospital for patients living at home did not decrease in 1998-2007 (Table 3). Stepwise adjustment (data not shown) of these unadjusted odds ratios showed no factors altered this trend. In our final model, the chance of dying out of hospital of those living at home was higher for cancer patients, those aged 85 or older, living in a multi-person household, living in moderate or low urbanized areas and in health care regions with less hospital beds. (Table 4)

**Table 3:** Unadjusted and adjusted trends in chance of dying at home and in care homes of patients (aged ≥65 y) who died of chronic life-limiting conditions 1998-2007

	1998	1999	2000	2001	2002	2003	2004	2005	2006	2007
PATIENTS LIVING AT HOME (N = 189 884)	OR of Home Death vs. Hospital Death (99% CI)									
Unadjusted odds ratios					NS*					
Adjusted odds ratios†					NS*					

PATIENTS LIVING IN CARE HOMES (N = 79 846)	OR of Care Home Death vs. Hospital Death (99% CI)									
Unadjusted odds ratios	Ref	1.14 (1.03-1.25)	1.19 (1.08-1.31)	1.24 (1.13-1.36)	1.44 (1.31-1.59)	1.47 (1.34-1.62)	1.46 (1.32-1.61)	1.50 (1.36-1.66)	1.52 (1.38-1.68)	1.65 (1.50-1.83)
Adjusted odds ratios‡	Ref	*1.08 (0.98-1.19)*	*1.06 (0.96-1.17)*	*1.05 (0.95-1.17)*	1.17 (1.05-1.31)	1.15 (1.03-1.29)	*1.11 (0.99-1.25)*	1.15 (1.03-1.30)	1.14 (1.01-1.29)	1.21 (1.07-1.37)

* The variable Year of Death was not selected in the forward stepwise likelihood ratio selection procedure of variables in the logistic regression analysis

† Odds ratios adjusted for underlying cause of death, age, sex, education, living arrangement, urbanization level and available hospital beds. Odds ratios of covariates are reported in Table 4.

‡ Odds ratios adjusted for underlying cause of death, age, sex, education, urbanization level, available residential beds and skilled nursing beds in care homes, and available hospital beds. Odds ratios of covariates are reported in Table 4.

**Table 4:** Adjusted odds ratio's of covariates in the logistic regression models of home death versus hospital death and care home death versus hospital death (table 3) of patients (aged ≥ 65 y) who died of chronic life-limiting conditions 1998-2007

	PATIENTS LIVING AT HOME (N = 189 884)	PATIENTS LIVING IN CARE HOMES (N = 79 846)
	
	AOR of Home Death vs. Hospital Death (99% CI)	AOR of Care Home Death vs. Hospital Death (99% CI)
CAUSE OF DEATH		
Cancer	Reference	Reference
Non-cancer	0.86 (0.83-0.88)	1.09 (1.04-1.15)
AGE		
65-74 year	Reference	Reference
75-84 year	*0.99 (0.96-1.02)*	1.51 (1.40-1.64)
85+ year	1.26 (1.22-1.31)	2.18 (2.01-2.36)
SEX	NS*	
Male		Reference
Female		1.13 (1.09-1.18)
EDUCATION	NS*	
Low-unknown/other		1.19 (1.09-1.29)
Higher secondary/Higher		Reference
LIVING ARRANGEMENT		†
Living alone	Reference	
Multi-person Household	1.95 (1.89-2.01)	
URBANIZATION		NS*
Very Strong/Strong	Reference	
Moderate/Low	1.42 (1.38-1.46)	
RESIDENTIAL BEDS IN CARE HOMES (Continuous per 1 bed/1000 ≥ 65 )	†	0.997 (0.996-0.999)
NURSING BEDS IN CARE HOMES (Continuous per 1 bed/1000 ≥ 65)	†	1.015 (1.012-1.018)
HOSPITAL BEDS (Continuous per 1 bed/1000)	0.896 (0.884-0.910)	1.033 (1.006-1.061)

*: Not significant. The variable was not selected in the forward stepwise likelihood ratio selection procedure of variables in the logistic regression analysis

†: Variable was not included in the model

Care home death was more likely in the period 1999-2001 compared with 1998, in 2002-2006 compared with previous years and again in 2007 compared with all previous years. (Table 3) This unadjusted trend of decreasing chances of care home residents dying in hospital was adjusted significantly by the increasing availability of skilled nursing home beds in care homes (data not shown). The other variables did not adjust the trend of the decreasing odds of dying in hospital. In our final model, the chance of dying out of hospital of those living in care homes was still more likely in each year of the study period compared to 1998, except in 1999-2001 and 2004, (Table 3) and was more likely for those who died from non-cancer conditions, with a higher age, females, having a lower or unknown educational attainment and living in healthcare regions with less residential beds and more skilled nursing beds in care homes and more hospital beds.(Table 4)

**Figure 4:**
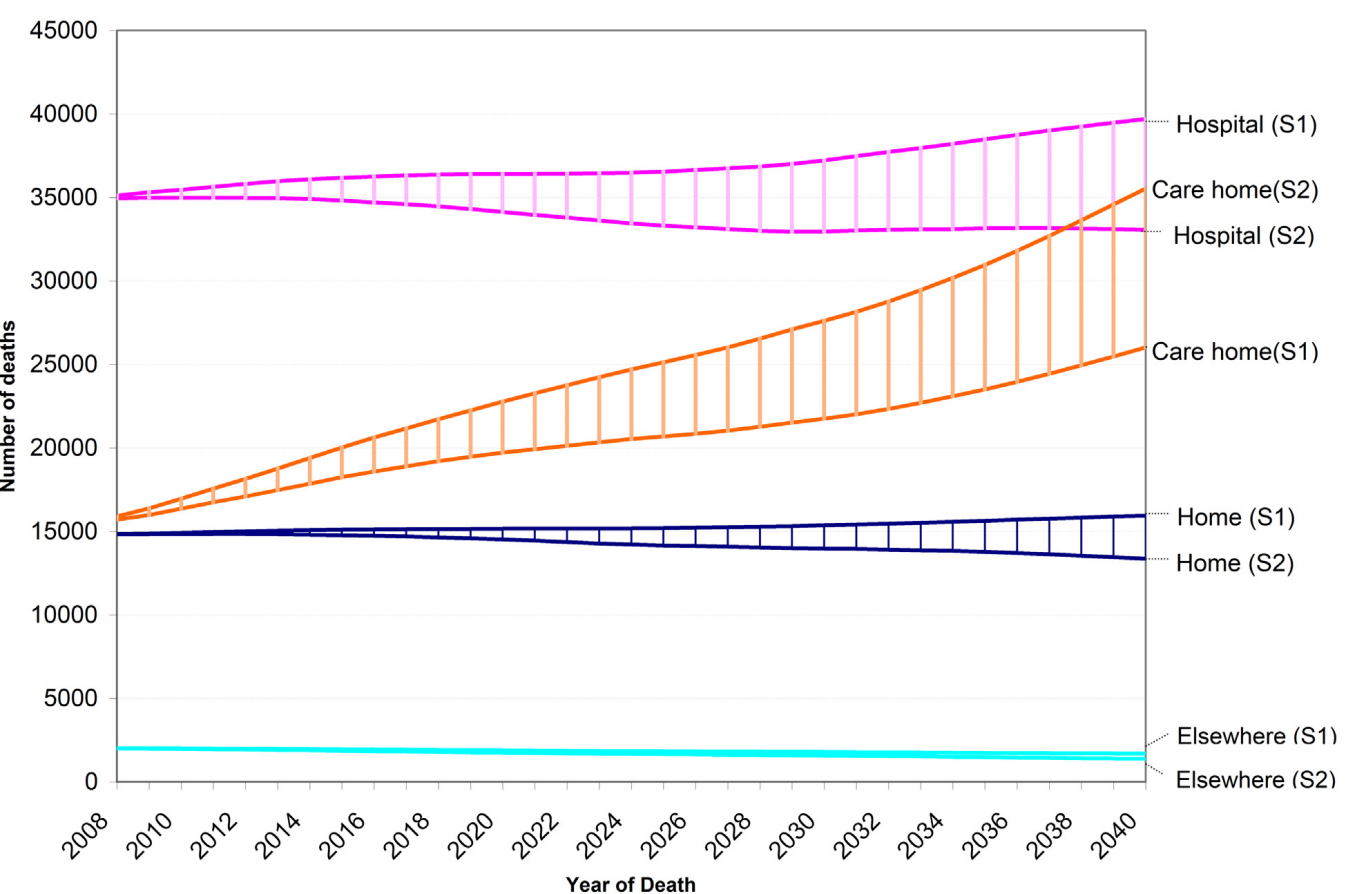
**Projected trends in proportion of home deaths, hospital deaths and care home deaths (2008-2040), based on 2 scenarios**. Age- and sex-specific mortality projections of the Belgian National Planning Bureau were used to perform linear projections of numbers of deaths from 2008 to 2040 in all care settings starting from the age- and sex-specific proportions of deaths in the different care settings in the period 1998-2007. Two scenarios were considered. In scenario 1 (S1) future trends in place of death were projected starting from the age- and sex- specific distribution of place of death in 2007. In scenario 2 (S2) the projection was based on the age- and sex-specific percentage point change in place of death from 1998 to 2007.

Mortality (aged ≥ 1 year) is expected to rise from 65 435 in 2007 to 83 388 in 2040. The first scenario (S1), in which the age-and sex-specific distribution of place of death of 2007 was projected, shows an increase of both care home (from 14 792 in 2007 to 26 030 in 2040) and hospital deaths (from 33 856 to 39 714) (Figure 4). The number of deaths at home would rise slightly from 14 726 to 15 954). The second scenario (S2), for which we projected the 10 year trends forward to 2040, shows an increase in care home deaths to 35 545. Deaths at home and in hospital show a slight decrease to 13 380 and 33 076. In 2038, the proportion of care home deaths (41.15%) would exceed that of hospital deaths (40.53%) (data not shown). The proportion of deaths at home would drop from 22.5% in 2007 to 16.05% in 2040.

## Discussion

In Belgium in the period 1998-2007 the proportion of deaths in hospitals decreased and in care homes increased, while that of home deaths remained stable. The proportion of care home residents dying in hospitals dropped from 31.0% in 1998 to 21.5% in 2007. The trend of declining chances of dying in hospital of care home residents was largely related to the substitution of residential beds by skilled nursing beds in care homes. The odds of dying in hospital of those living at home did not diminish during the study period. Continuation of the trends in place of death during 1998-2007 would lead to more than double the number of care home deaths by 2040 and to a larger proportion of deaths in care homes than in hospitals.

For the first time trends in place of death were studied and related not only to shifts in underlying cause of death, age and sex of the deceased population, but also to changes in living arrangements, educational attainment, urbanization levels of the place of residence and availability of beds in hospitals and care homes. Trends in place of death proved to be related to changes in care levels in care homes. In contrast with previous research, our study considered all patients in all care settings over a 10 year period. Trends in place of death have not been studied in continental Europe before. Potential limitations of this study are connected to the use of death certificate data: possible unreliability of the certification and coding of some underlying diseases and sociodemographic information, missing values for variables related to the background of the deceased and the lack of information on factors which affect place of death [28,29]. Multilevel modelling may have been an alternative appropriate approach to analyze data related to individuals and geographical areas.

Patterns in the previous century showed an increasing hospitalization of death. Concentration of health care in hospitals and the reduced availability of informal care givers due to sociodemographic and economic developments lead to a growing number of hospital deaths, and the proportion of deaths occurring at home decreased from more than half to a quarter or less [30,31]. From the 1980s onwards a decrease in hospital deaths was observed in many countries, both in cancer and non-cancer patients [11,12,14,15,17,20,21,23,32]. In many countries, this trend was not balanced by an increase in home deaths [18-20], even in cancer patients [11,14,15], because of the ongoing sociodemographic and economic dynamics that caused the hospitalization of death trend in the first part of the century, and the growing availability of other care options for end-of-life care, hospices in some countries and care homes in others. In consequence the proportion of care home deaths in the UK, Canada, USA, and Australia increased in the last twenty years of the previous century by 4% to 10% [11,17,20,21,23]. In our findings additional care options in care homes supported shifts in place of death in Belgium. The additional availability of skilled nursing care beds in care homes replacing residential beds resulted in a considerable growth of care home deaths, especially among existing residents. The latter is not obvious, in light of the large-scale transfer of care home residents to hospital at the end of life observed previously [33,34]. Because of the shift towards skilled nursing care beds the odds of care home residents dying in hospital decreased drastically in a period of only ten years. The trend of decreasing hospital death risk of care home residents remained significant after adjusting for available skilled nursing beds in care homes. Therefore this trend is not just a consequence of an increase in skilled nursing resources but probably also of the development of palliative care in care homes. From 1997 onwards in-house palliative care reference persons responsible for supporting, coaching and educating care givers were appointed [9]. Involvement of these palliative care reference persons is probably associated with reduced odds of dying in hospital of care home residents since supporting out-of-hospital death is one of there main objectives [9] and with the rapid development of policies on advance care planning in Flemish care homes from 2000 onwards, resulting in a do-not-hospitalize option available in 90% of care homes by 2006 [35].

The implications of our findings are considerable. Between 1998 and 2007 an increase of more than 20 000 skilled nursing beds in care homes, replacing residential beds, succeeded in reducing the hospitalization risk of care home residents and to some extent of older patients living at home. Further reducing that risk during the coming decades or even maintaining it at the 2007 level, in the context of a rapidly ageing population and a rapidly growing number of deaths, will require a massive investment in skilled nursing home beds and other end-of-life care options outside hospitals; care homes could become the central location for end-of-life care in Belgium in the future, replacing private residences in the first part of the 20^th ^century and hospitals thereafter. The economic implications of this trend are very important and should be considered. Governments should provide sufficient skilled nursing resources in care homes to respect patients' preferences and needs.

Even if the facilities are present, providing quality end-of-life care in care homes will require a palliative care approach and additional investment in palliative care services as treatment of pain and symptoms is often poor and communication about the needs and preferences of terminally ill care home residents is inadequate, due to insufficient staffing, lack of time and resources and inadequate financial reimbursement of palliative care costs from healthcare insurance [36-38]. Future research should examine if and how quality of care at the end of life has changed with the changing place of death.

## Conclusions

We found that in Belgium between 1998 and 2007 the proportion of deaths in hospitals decreased (-3.4%) and increased in care homes (+4.3%), while that of home deaths remained stable. This trend was largely explained by the proportion of care home residents dying in hospitals dropping from 31% to 21.5%, a shift strongly related to the substitution of residential beds by skilled nursing beds in care homes. Continuation of this trend would lead to more than double the number of care home deaths by 2040. The implications of our findings are considerable. Care homes may become the main locus of end-of-life care in the future and governments should provide sufficient skilled nursing resources in care homes to fulfil the preferences and needs of their residents.

## Authors' contributions

DH and JC performed the statistical analyses and drafted the manuscript. All authors participated in the conception and design of the study, the acquisition of data, the interpretation of data, revising the manuscript critically for important intellectual content, and have given final approval of the version of the manuscript to be published.

## Declaration of competing interests

The authors declare that they have no competing interests.

## Pre-publication history

The pre-publication history for this paper can be accessed here:

http://www.biomedcentral.com/1471-2458/11/228/prepub
